# Alleviation of Cadmium Stress by Silicon Supplementation in Peas by the Modulation of Morpho-Physio-Biochemical Variables and Health Risk Assessment

**DOI:** 10.3390/life12101479

**Published:** 2022-09-23

**Authors:** Tahira Batool, Sabiha Javied, Kamran Ashraf, Khawar Sultan, Qamar uz Zaman, Fasih Ullah Haider

**Affiliations:** 1 Department of Environmental Sciences, University of Lahore, Lahore 54590, Pakistan; 2 Department of Food Sciences, Government College University, Faisalabad, Sahiwal Campus, Sahiwal 57000, Pakistan; 3 Key Laboratory of Vegetation Restoration and Management of Degraded Ecosystems, South China Botanical Garden, Chinese Academy of Sciences, Guangzhou 510650, China; 4 University of Chinese Academy of Sciences, Beijing 100039, China

**Keywords:** biochemical, cadmium, food chain, growth, membrane leakage, peas, stress

## Abstract

Agricultural soil quality degradation by potentially toxic elements, specifically cadmium (Cd), poses a significant threat to plant growth and the health of humans. However, the supplementation of various salts of silicon (Si) to mitigate the adverse effect of Cd on the productivity of peas (*Pisum sativum* L.) is less known. Therefore, the present investigation was designed to evaluate the exogenous application at various levels (0, 0.50, 1.00 and 1.50 mM) of silicate compounds (sodium and potassium silicates) on pea growth, gaseous exchange, antioxidant enzyme activities and the potential health risk of Cd stress (20 mg kg^−1^ of soil) using CdCl_2_. The findings of the study showed that Cd stress significantly reduced growth, the fresh and dry biomass of roots and shoots and chlorophyll content. In addition, electrolyte leakage, antioxidant enzymes and the content of Cd in plant tissues were enhanced in Cd-induced stressed plants. An application of Si enhanced the development of stressed plants by modulating the growth of fresh and dry biomass, improving the chlorophyll contents and decreasing leakage from the plasma membrane. Furthermore, Si addition performed a vital function in relieving the effects of Cd stress by stimulating antioxidant potential. Hence, a significant level of metal protection was achieved by 1.00 mM of potassium silicate application under the Cd levels related to stress conditions, pointing to the fact that the Si concentration required for plant growth under Cd stress surpassed that which was required for general growth, enzymatic antioxidants regulation and limiting toxic metal uptake in plant tissues under normal conditions. The findings of this research work provide a feasible approach to reduce Cd toxicity in peas and to manage the entry and accumulation of Cd in food crops.

## 1. Introduction

Soil is a fundamental supporting medium in terrestrial ecological systems, serves as a main sustainable source of nutrients accessible to plants for growth [1] and provides conditions under which the diffusion of materials and energy takes place. Soil contains heavy metals (HMs), including cadmium (Cd), arsenic (As), lead (Pb) and mercury (Hg), that may build up in the environment and endanger humans and other living things on the earth [2]. The HM contamination of soil is a significant threat aggravated by human activities and operations, including wastewater and sewage sludge usage for irrigation purposes, the mining of minerals, fertilizer, pesticide use and increasing vehicle and industrial emissions [1,2,3]. Plant HM contamination leads to changes in morphology, physiology, biochemistry and ultrastructure [4]. Heavy metals that are relatively mobile in soil include Cd, which is highly harmful to both plants and animals [5,6]. Through the food chain, cadmium gradually accumulates in humans and causes serious health disorders [7,8,9,10,11]. Due to cadmium toxicity, plants experience negative impacts, including growth suppression, leaf chlorosis, the lack of photosynthetic pigments, metabolic disruption, oxidative stress, homeostasis disorder and decreased agricultural production [1,2].

In terms of providing food and forage for humans, legumes are a broad category of plants that come in second after cereals [12]. The pea (*Pisum sativum*) is a valued legume crop that is cultivated extensively around the world for its flavor and nutritional benefits [13]. The quality of peas for human and animal use has suffered because of the massive influx of Cd into pea fields brought on by the excessive use of fertilizers. The uptake of Cd by plants is linked to numerous health hazards, such as kidney, bone and lung disease [8,12]. Before reaching plant roots, Cd movement into plants occurs in root cortical tissues and then makes its way into the xylem through a symplastic and/or apoplastic pathway that ultimately reaches grain, which is very toxic for human consumption [14,15,16]. Plant species vary in their capacity to absorb Cd through the xylem and phloem, making the roots an important organ for this process. In peas, tap root development, photosynthesis and the uptake of other macro or micro-nutrients are all negatively impacted by Cd after its absorption [17]. Therefore, it is vital to take precautions to prevent Cd from being absorbed by plants and to inhibit its entry into grains [18]. We need to learn more about how plants adapt to their environment, and one way to do that is to identify the inorganic and organic amendments that facilitate stress tolerance.

For the reclamation of metal-contaminated soil, various eco-friendly and cost-effective approaches have been explored, including biochar, organic acids, farmyard animal waste, nanomaterials, etc. [19,20,21,22,23]. Silicon (Si) usage increases plant responses to metal toxicity with promising outcomes [24]. To cope with abiotic stresses, silicon (Si) should be utilized in bioavailable forms, as it may be abundant in soils but usually not bioavailable to plants when cultivated in Cd-contaminated soils [25]. According to several studies, the reduction in Cd uptake and movement among plant organs in crops such as cotton (*Gossypium herbaceum*) [26], cabbage (*Brassica oleracea*) [27], wheat (*Triticum aestivum*) [28], peanuts (*Arachis hypogaea*) [29] and rice (*Oryza sativa*) [30] can be mostly ascribed to Si. In another study, rice grains and straw that were treated with calcium silicate had less Cd in them [31].

Previous studies on Si have often used only a single dose of one type of Si salt in cereals [1,2]. The use of different Si salts at varying levels of concentration is not well studied in legumes crops, especially in peas. The negative effects of Cd concentration in dicots varies because of the interruption of the absorption of essential nutrients through reduced root proliferation as compared to monocots having a fibrous root system. Therefore, the role of Si in regulating Cd varies depending upon the root system. Based on the literature review, it was found that no study has been conducted yet to investigate the effectiveness of Si compounds at ideal concentrations for peas and the entry of Cd into the human body system through pea consumption. To this end, this experimental approach hypothesizes that foliar-applied Si salts minimize Cd uptake by reducing Cd bioavailability in the soil. The main objectives of this research work were to: (1) assess the effects of various silicate salts (sodium silicate and potassium silicate) at various concentration ranges on the growth, physio-biochemical and enzymatic antioxidant variables in Cd-spiked soil material; (2) evaluate the effectiveness of silicate salts for limiting Cd stress and the accumulation of Cd in pea plants; and (3) evaluate the health risk in children and adults of local communities.

## 2. Materials and Methods

### 2.1. Study Site, Experimental Design and Treatments

This research work involved a pot experiment that was conducted from the first week of November, 2021, to the last week of December, 2021, in a greenhouse at the Environmental Science Department, The University of Lahore (31.38812° N, 74.24135° E), Pakistan. Experimental treatments comprised three factors: silicon compounds (sodium silicate [Na_2_SiO_3_], and potassium silicate [K_2_O_3_Si]), the application of a range of doses (0, 0.50, 1.00 and 1.50 mM) and Cd stress (control and cadmium stress 20 mg kg^−1^ using CdCl_2_) procured from Sigma Chemical Co. (St. Louis, MO, USA) using a completely randomized design (CRD) under the factorial arrangement (every replication consisted of three pots per treatment).

### 2.2. Crop Management

Soil samples (sandy clay loam) were collected at a depth of 0–30 cm (from the study site), air dried and then passed through a 5 mm sieve. The initial physico-chemical attributes of the soil used in the study were pH 7.1, EC 1.76 dS m^−1^ and SAR 45.4 (mmol L^−1^)^1/2^. Bicarbonates were 0.12 mg L^−1^, and carbonates were absent. The soil (7 kg of soil per pot) in this experiment was first passed through a 5 mm size sieve and was later filled into plastic pots with appropriate volumetric dimensions (upper diameter size of 22.5 cm, base diameter size of 16.5 cm and depth of 18 cm). A pea variety plant with indeterminate growth characteristics, “Sarsabz”, having an excellent germination capacity (98%), was used, procured from the Vegetable Section, Ayyub Agricultural Research Institute, Faisalabad, Pakistan. Ten healthy seeds were directly sown, grown and maintained in every soil pot throughout the experiment on 7 November 2021. Seven seedlings of equivalent size were kept up per pot after washing with distilled water. In the early stages, metal was not included, and metal stress application began after a 14-day development period. After this period, the seedlings had four to five leaves. After that, metal stress was applied using a solution form, and metal stress application was based on previous studies [2,32,33,34]. Just before the application of the Cd solution, as well as after 2 days, manual hoeing was performed for the homogenization of Cd contamination. The plants, which were kept free of metal stress (control plants), were only supplied with normal nutrient solutions. A Hoagland solution (50%) consisting of moderate strength was applied to cater to the nutritional needs of plants at a rate of 1 L per week per pot. Based on the physical observations of the plants in pots, the necessary agronomic practices such as irrigation and weeding were carried out regularly as required during the experiment. Foliar sprays of silicate salts were carried out over 15 days of stress application from 10:00 am until 11:00 am on seedlings, and the treatments were carried out only once throughout the complete experiment [35]. Upon completion of a 15-day period involving the application of Si treatment, pea plants were carefully taken out of pots, observed and evaluated by measuring various selected parameters on 28 December 2021.

### 2.3. Morphological, Phenological and Biomass Variables

From each replication, a set of five plant samples were picked randomly and harvested at the completion of 40 days after the seeding (DAS) period. After counting the leaves, plant samples were washed with distilled pure water, and the height of the plant and the shoot and root length were measured with a meter rod. Plant biomass consisting of roots and shoots was analyzed soon after harvesting, and the dry biomass of the sample was recorded after oven drying (70 °C for 72 h) by using a weighing balance. A lightweight leaf area instrument (LI-3000, LI-COR Lincoln, NE, USA) and a non-destructive method were used to determine the leaf area of a plant [36].

### 2.4. Physio-Biochemical and Water-Related Variables

On the 45 DAS, the fully developed uppermost plant leaves were selected from 9:00 a.m. to 12:00 noon for measuring the photosynthetic rate (*A*), stomatal conductance (*gs*), transpiration rate (*E*) and SPAD chlorophyll contents by utilizing an SPAD-502 chlorophyll meter (KONICA MINOLTA, Tokyo, Japan) and a portable photosynthesis infrared gas analyzer (IRGA, Analytical Development Company, London, UK). A leaf of constant size from each treatment was used to calculate the relative water content (RWC), as described by using the following equation: RWC% = (fresh weight − oven-dried weight/fully turgid weight − oven-dried weight) × 100, and electrolyte leakage (EL) was calculated with the help of the formula described by [37]: EL% = [EC1/EC2] ×100, where EC1 = EC of the solution containing leaves in test tubes, and EC2 = EC of the solution in test tubes after autoclaving for 20 min at 121 °C.

### 2.5. Enzymatic Antioxidant Variables

Fresh leaf samples (1.0 g) were extracted by using phosphate buffer (50 mM, pH~7.8), and the resulting homogenate was centrifuged (15,000× *g* for 10 min). The supernatant thus obtained was utilized for analyzing enzyme activity. The peroxidase (POD) activity was determined following the technique presented by Velikova et al. [38], and the catalase activity (CAT) was measured according to the method described by Aebi [39]. The superoxide dismutase activity (SOD) was assayed following the procedure presented by Beauchamp and Fridovich, [40], and they reported a method of superoxide dismutase (SOD) activity measurement, which was followed in this research work.

### 2.6. Cadmium Contents in Plant Tissues

A mixture of di-acid of a certain proportion (HNO_3_:HClO_4_ at 2:1) of analytical grade quality was used to determine the concentration of Cd in various parts of pea plants, such as shoots, roots and grains. For analytical measurements of the substance of Cd^2+^, an atomic absorption spectrophotometer instrument (Perkin-Elmer, Model 3300, Waltham, MA, USA) was equipped with an air/acetylene burner head, and a nebulizer kit was used according to the procedure mentioned by Ryan [41].

### 2.7. Health Risk Assessment

An assessment of the Health Risk Index (HRI) for Cd in all samples was carried out in this study. The HRI value was determined by calculating the daily intake of metal (DIM) first and then by dividing this value by an oral reference dose (RfD) of Cd in vegetables [42]. An established RfD value of Cd was found to be 0.001 mg kg^−1^ body weight per day [1]. The estimation of the daily intakes of Cd by the local population was calculated by multiplying the levels of Cd in grains by the daily food intake of a person and the conversion factor and by dividing by the average body weight of an individual. The average body weight in Pakistan of adults 18–60 years is 65 kg, and for children, it is taken as 35 kg for age 7–17 years. The average vegetable consumption per capita is 0.15 kg day^−1^ [43]. DIM = (Cd in grain × daily food intake of vegetable × conversion factor) divided by the average body weight. It is established knowledge that an HRI index value of (>1) is unsafe for humans [1,44].

### 2.8. Statistical Analysis

The data collected in the experiment was processed by employing Fisher’s Analysis of Variance (ANOVA) method, which mainly helped in the significance testing of numbers. As part of the statistical analysis, the Highest Significant Difference (HSD) test was used for the comparison of means, where the ANOVA showed significant differences. All data processing and statistical calculations were carried out using statistical software (Statistix, Version 10), and for graphical work, Microsoft Excel (2013 version) (Microsoft Corporation, Redmond, WA, USA) was used in this study.

## 3. Results

### 3.1. Morphological, Phenological and Biomass Variables

**Figure 1:**
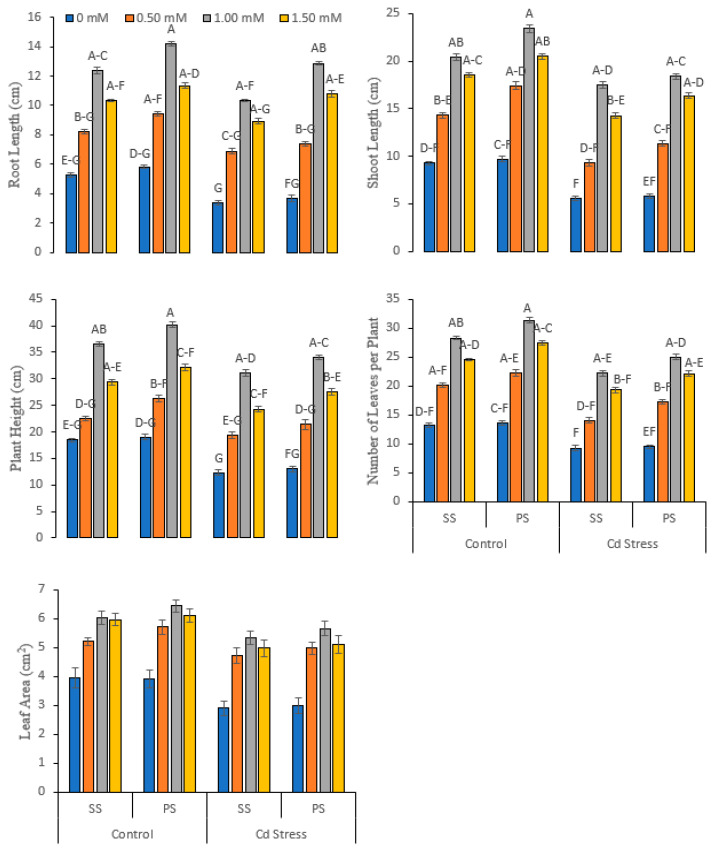
Growth and phenological attributes of peas under various foliar applied treatments (0.00, 0.50, 1.00 and 1.50 mM) of silicon (Si) salts (sodium silicate (SS) and potassium silicate (PS) grown under normal (0 mg kg^−1^) and cadmium (Cd)-stressed conditions (20 mg kg^−1^). The different letters on the bars show significant differences across treatment means at *p* ≤ 0.05; values denote the means ± SD and replicated thrice.

**Figure 2:**
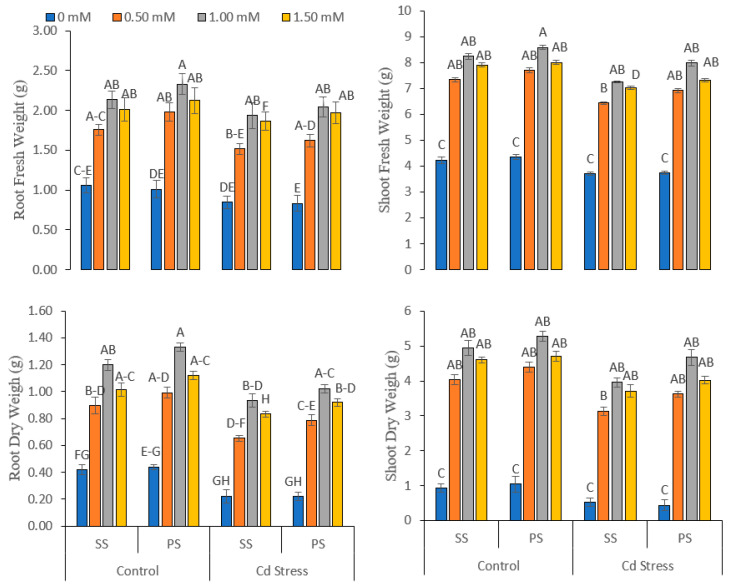
Fresh and dry biomass of roots and shoots of peas under various foliar applied treatments (0.00, 0.50, 1.00 and 1.50 mM) of silicon (Si) salts (sodium silicate (SS) and potassium silicate (PS)) grown under normal (0 mg kg^−1^) and cadmium (Cd)-stressed conditions (20 mg kg^−1^). The different letters on the bars show significant differences across treatment means at *p* ≤ 0.05; values denote the means ± SD and replicated thrice.

Cadmium stress (Cd), various silicate chemicals (SC), different doses (L) of foliar applied Si and their interaction effect (Cd × SC × L) profoundly affected (significance, *p* ≤ 0.01) the morphological, phenological and biomass variables of pea plant development under normal (0 mg kg^−1^ Cd) and Cd-stressed conditions (20 mg kg^−1^ Cd). Cadmium stress decreased root length (16.61%), shoot length (26.29%), plant height (18.49%), the number of leaves (23.37%), leaf area (15.31%), root fresh weight (12.22%), shoot fresh weight (10.51%), root dry weight (24.73%) and shoot dry weight (19.52%) in comparison with the control (Figure 1 and Figure 2).

A higher increase was registered in root length (14.51% and 25.24%), shoot length (14.70% and 5.14%), plant height (9.83% and 9.64%), the number of leaves (10.95% and 12.61%), leaf area (6.78% and 6.57%), root fresh weight (9.38% and 5.67%), shoot fresh weight (4.12% and 9.91%), root dry weight (10.83% and 9.67%) and shoot dry weight (6.88% and 18.22%), respectively, where the foliar application of 1.00 mM concentration of potassium silicate solution was applied as compared to sodium silicate under normal and Cd-stressed conditions (Figure 1 and Figure 2).

### 3.2. Physio-Biochemical and Water-Related Variables

**Figure 3:**
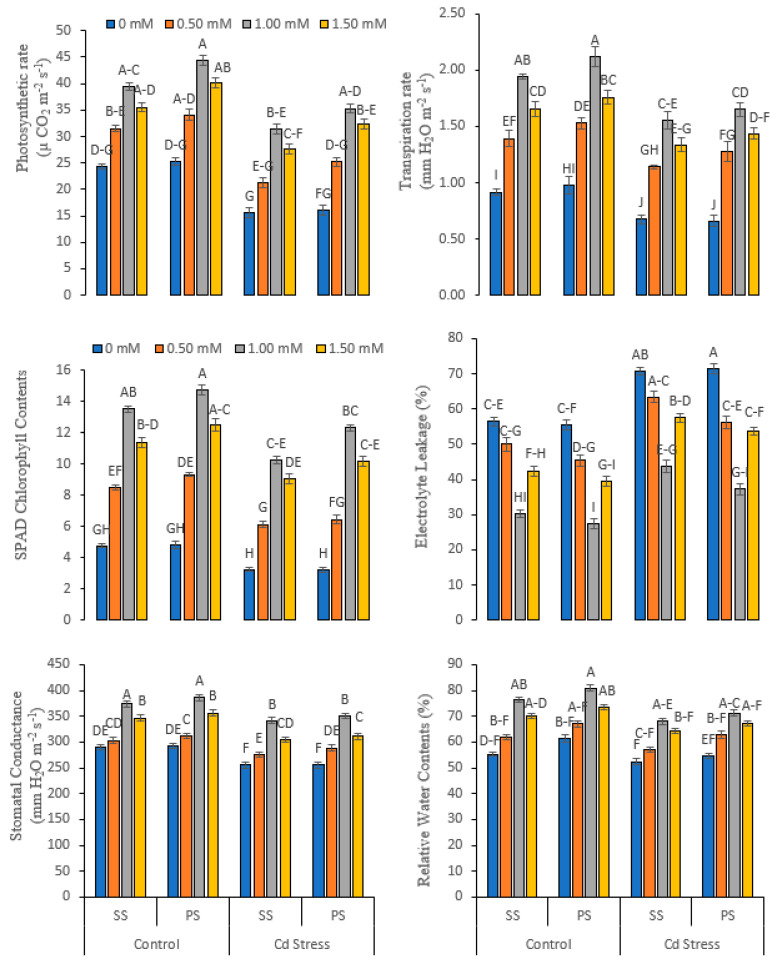
Physio-biochemical and water-related variables of peas under various foliar applied treatments (0.00, 0.50, 1.00 and 1.50 mM) of silicon (Si) salts (sodium silicate (SS) and potassium silicate (PS)) grown under normal (0 mg kg^−1^) and cadmium (Cd)-stressed conditions (20 mg kg^−1^). The different letters on the bars show significant differences across treatment means at *p* ≤ 0.05; values denote the means ± SD and replicated thrice.

Various doses of foliar-applied silicate salts and the cadmium content interaction effect (Cd × SC × L) significantly (*p* ≤ 0.01) affected the physio-biochemical variables of pea plants grown under normal and Cd-stressed conditions. Variations were observed in the physio-biochemical attributes (i.e., photosynthetic rate (*A*), stomata conductance (*gs*), transpiration rate (*E*), chlorophyll content, electrolyte leakage and relative water content as affected by Cd stress (Figure 3)). The range of the photosynthetic rate was (15.67–44.40 µ CO_2_ m^−2^ s^−1^), the rate of transpiration was (0.66–2.12 mm H_2_O m^−2^ s^−1^), the stomatal conductance was (255.33–385.70 mm H_2_O m^−2^ s^−1^), the chlorophyll content was (3.24–14.75), the electrolyte leakage was (27.44–71.37%) and the relative water content was (52.44–80.90%). The minimum value was noted in Cd-stressed plants. In comparison to the non-stressed control, Cd stress reduced the photosynthetic rate (33.94%), the rate of transpiration (26.22%), stomatal conductance (10.34%), chlorophyll content (23.24%), electrolyte leakage (30.79%) and relative water content (9.65%). The resilience and capacity against the Cd stress were improved by Si supplementation. The highest influences in all the physio-biochemical attributes were noticed in 1.00 mM Si supplementation using potassium silicate under Cd stress (Figure 3).

### 3.3. Enzymatic Antioxidants Variables

**Figure 4:**
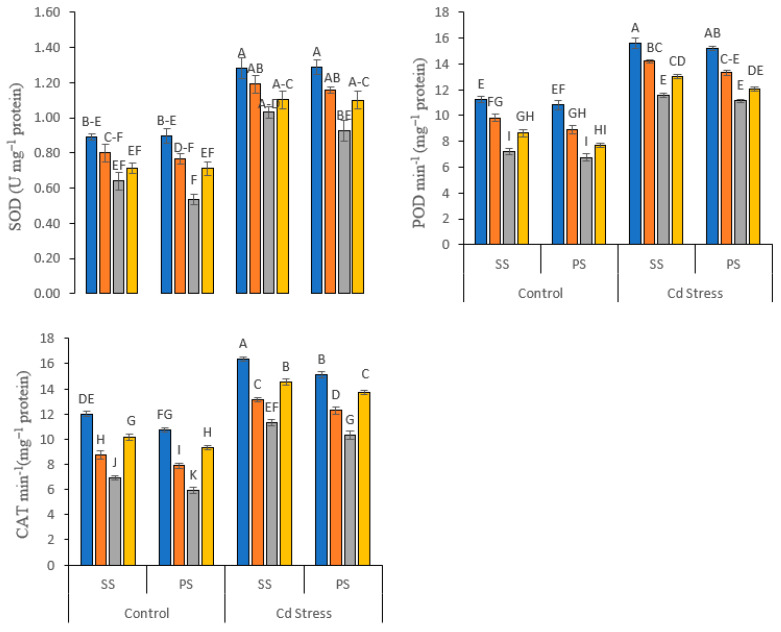
Enzymatic antioxidant attributes of peas under various foliar applied treatments (0.00, 0.50, 1.00 and 1.50 mM) of silicon (Si) salts (sodium silicate (SS) and potassium silicate (PS)) grown under normal (0 mg kg^−1^) and cadmium (Cd)-stressed conditions (20 mg kg^−1^). The different letters on the bars show significant differences across treatment means at *p* ≤ 0.05; values denote the means ± SD and replicated thrice.

All the enzymatic activities in Cd-stressed plants were stimulated in comparison to the non-stressed control (Figure 4). The highest enzyme activities under Cd stress were increased by 52.70% for SOD, by 49.44% for POD and by 32.88% for CAT as compared to the pots where no Cd stress was applied. In addition, the exogenous application of 1.00 mM Si in the form of potassium silicate improved the SOD, POD and CAT activities by 2.68%, 5.00% and 7.08%, respectively, in comparison to sodium silicate under Cd stress. Potassium silicate proved to have a better response as compared to sodium silicate (Figure 4).

### 3.4. Cadmium Contents in Plant Tissues

**Figure 5:**
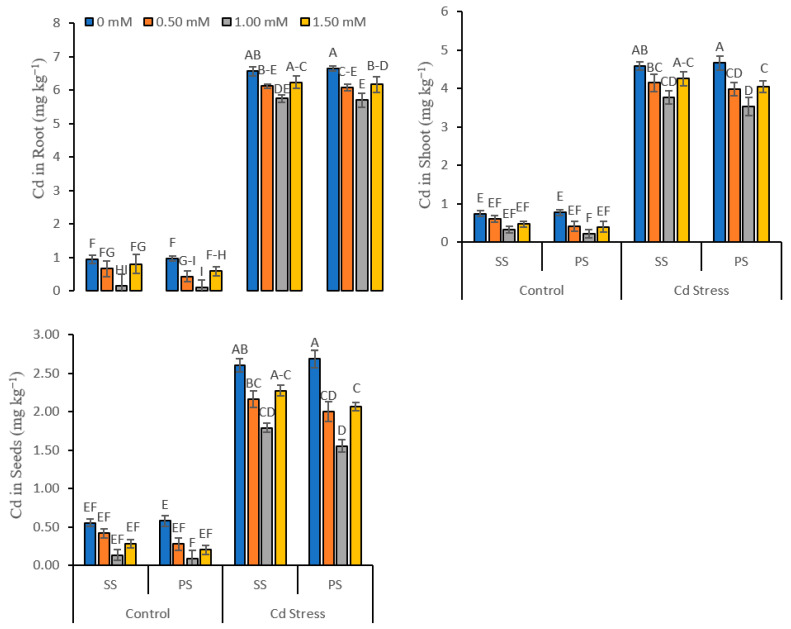
Cadmium concentration in various plant parts of peas under various foliar applied treatments (0.00, 0.50, 1.00 and 1.50 mM) of silicon (Si) salts (sodium silicate (SS) and potassium silicate (PS)) grown under normal (0 mg kg^−1^) and cadmium (Cd)-stressed conditions (20 mg kg^−1^). The different letters on the bars show significant differences across treatment means at *p* ≤ 0.05; values denote the means ± SD and replicated thrice.

Cadmium stress, various silicate salts, different doses of foliar-applied Si and their interaction effect (Cd × SC × L) significantly (*p* ≤ 0.01) affected the Cd contents in the roots, shoots and grains of peas plants grown under normal and Cd-stressed conditions (Figure 5). Cd stress increased the Cd contents in roots (962.03%), shoots (740.03%) and grains (568.32%) as compared to the control. The minimum Cd content was (0.16 mg kg^−1^) in roots, (0.32 mg kg^−1^) in shoots and (0.13 mg kg^−1^) in seeds for sodium silicate, and it was (0.11 mg kg^−1^) in roots, (0.21 mg kg^−1^) in shoots and (0.09 mg kg^−1^) in seeds for potassium silicate under controlled conditions. The minimum Cd content was (5.75 mg kg^−1^) in roots, (3.77 mg kg^−1^) in shoots and (1.79 mg kg^−1^) for sodium silicate, and it was (5.70 mg kg^−1^) in roots, (3.51 mg kg^−1^) in shoots and (1.55 mg kg^−1^) in grains for potassium silicate under Cd-stressed conditions, observed where the foliar application of a 1.00 mM solution was applied as compared to control (0 mM). Potassium silicate decreased Cd content (0.32%) in roots, (3.20%) in shoots and (5.88%) in seeds as compared to sodium silicate under Cd-stressed conditions. The decreasing pattern in terms of Cd content in roots for Cd stress was noted to be as follows: Cd stressed conditions > controlled conditions; for silicate chemicals, it was sodium silicate > potassium silicate; and for the Si treatment, it was 0 mM > 1.50 mM > 0.50 mM > 1.00 mM (Figure 5).

### 3.5. Health Risk Index

An analysis of the values of the Cd of HRI by the food chain for children and adults showed that there was a decrease in metal uptake and a resultant bioaccumulation by the application of Si salts in comparison with the control sample (Table 1). In the case of adults, for instance, the HRI values varied between 0.03 and 0.53, and for the children, it varied from 0.03 to 0.98 as the highest value for the control and the lowest value for the Si concentration (1.00 mM) in the form of potassium silicate.

### 3.6. Correlation and Principal Component Analysis

**Figure 6:**
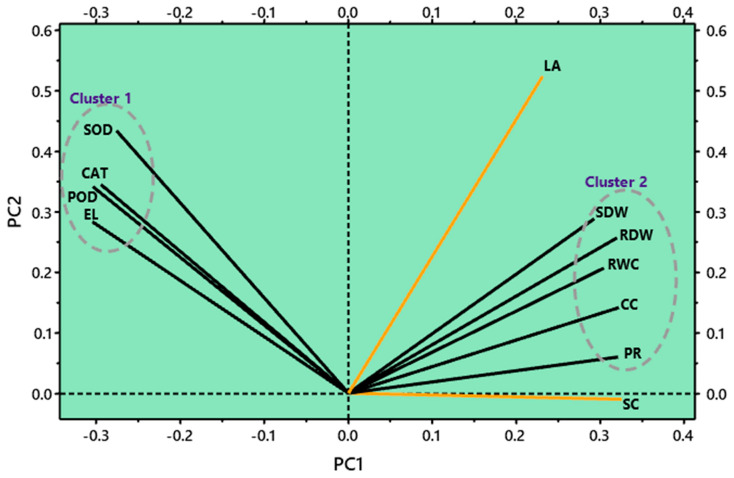
Principal component analysis plot representing correlations among variables and clusters for Cd stress and foliar-applied Si levels in peas. RWC = Relative water content; CC = Chlorophyll content; LA = Leaf area; SDW = Shoot dry weight; RDW = Root dry weight; SOD = Superoxide dismutase activity; POD = Peroxidase activity; CAT = Catalase activity; EL = Electrolyte leakage; PR = Photosynthetic rate; SC = Stomatal conductance.

To substantiate the validity of findings in peas plants, the growth, enzymatic, water-related and biochemical attributes were checked for positive and negative associations (Table 2). All the enzymatic attributes (SOD, POD and CAT) along with the electrolyte leakage were negatively correlated with chlorophyll contents, shoot dry weight, root dry weight and relative water content. Electrolyte leakage correlated negatively with shoot dry weight, root dry weight and relative water contents and were significantly positively correlated with POD, SOD and CAT activities. The chlorophyll contents were positively correlated with shoot dry weight, root dry weight and relative water contents of pea plants after the application of Si under Cd-stressed conditions (Table 2). The results from the principal component analysis (PCA) plot represent a correlation among variables and clusters for Cd stress and foliar-applied Si levels in peas (Figure 6). From the observation of the study, the PCA revealed that the growth, water-related and physio-biochemical attributes were split into two main clusters. The first cluster of CAT, POD, EL and SOD is about the enzymatic and biochemical variables that are related to the stress response of plants when subjected to elevated levels of potentially toxic Cd metals in the pot experiment. The second cluster was formed by variables related to growth, physiological and water-related attributes. It seems that there is an inverse correlation between the cluster 1 variables and the SC.

## 4. Discussion

Agricultural soils polluted by elevated toxic metal contents such as Cd have led to a significant decrease in plant health, growth and productivity on a global level [45]. The findings of this research work prove that Cd-induced stress significantly lowered the growth, phenological and biomass (fresh and dry) related traits. However, this situation was reversed when Si was supplied in the form of foliar application at a rate of 1.00 mM. Similar observations have been noted by Guo et al. [46] and Heile et al. [2]. Plants undergoing metal-induced stress and the application of Si may promote the growth and development of plants in several ways, including enhancing chlorophyll content, increasing cell division, increasing nutrient levels, spreading root volume and releasing organic acids [47,48,49]. They stimulate plant growth by decreasing ROS generation and by improving the antioxidant defense mechanism [50,51]. However, structural changes enforced by Cd-content-related stress in peas are positively affected by Si in terms of plant integrity [46,48]. Si treatment plays a vital role in mitigating Cd-induced stress conditions in plants by improving the conditions for the growth of pea plants. The chemical reaction between Si and Cd elements causes an increase in the absorption of various essential elements in the tissues of a plant. It may be the cause of the healthy growth and development of crop plants under stressful situations [52]. The mineral nutrient content increase in Si-treated plants may also cause an increase in biomass [53].

Cadmium-induced stress reduces cellular activity and physiological functions such as the photosynthesis process, most likely due to an imbalance in nutritional needs, metallic toxicity and osmotic and oxidative stresses [54]. This research work has shown that Si application enhances abiotic stress alleviation (i.e., Cd-induced stress) by maintaining osmotic stress and nutritional balance in pea plants. Ming et al. [55] reported an improvement in the photosynthesis process by the addition of Si salt as potassium silicate, which is possibly linked to the biological processes involved in the mitigation of stress-related damages in plants. The rise in the capability of antioxidant defenses is alleviated by the oxidative damage of enzymes by Si addition, which helps in plant photosynthesis [1,2,54]. Similarly, the enhancement of Si in pea plants developed under Cd stress reduced the electrolyte leakage in the tissues of plants. This points to the fact that exogenously applied Si likely protects and promotes membrane stability under Cd-stressed conditions in pea plants [8,56]. The research work of Kim et al. [57] revealed that stress in plants gives rise to elevated levels of electrolyte leakage when there is a rise in linolenic acid levels and a lowering of linoleic acid, whereas these membrane-associated fatty acids are much less of a problem in terms of damage to plants in Si-treated conditions [47]. In this work, electrolyte leakage was observed to be higher under enriched Cd levels [1,2], but this effect was significantly reduced with the application of silicon. The most effective Si dose level was found to be 1.0 mM by using potassium silicate for the alleviation of Cd-induced stress.

Metal stress in plants causes serious metabolic and physio-biochemical disorders [58]. Hence, plants have developed specific types of responses to protect themselves from toxic metal stress apart from additional cross-stress responses [2]. In plant cells, ROS are generated at a normal pace under the general functioning of cellular metabolism, but when subjected to metal stress, an overproduction of ROS is commonly observed [8,28]. The production of ROS is mainly regulated by different types of antioxidants, enzymatic and non-enzymatic [59]. In our research, the actions of enzymatic antioxidants, including the SOD, POD and CAT, increased with metal-content-related stress. This rise was higher without Si addition in comparison to the control plant samples. However, the incorporation of Si at a concentration of 1.00 mM decreased the activities of enzymatic antioxidants up to a safer level, as explained in several studies [2]. According to the findings of Gong et al. [60], the addition of Si in soils decreased the activities of SOD, POD and CAT. For example, in alfalfa (Medicago sativa) plants, the activities of CAT and SOD were noted to be much higher in the roots of plants without Si application under Cd stress [56]. In this experiment, the POD activity was increased by Cd^2+^ stress, and the optimum level was observed to be at a concentration of 1.00 mM of Si application. Various observations have also been reported by researchers about the changing activity of POD with Si addition in plants experiencing oxidative stress [57]. There is an interesting work by Rios et al. [54], which reported that Si treatment created conditions in which there was a significant decrease in ROS generation and a surge in ROS scavenging of the antioxidants of enzymatic and non-enzymatic types. Hence, Si possibly reduces oxidative stress in plants, primarily caused by Cd content, at the scale of cellular functioning, most likely caused by the efficient utilization of metabolic pathways involving ROS scavenging, and it might also ameliorate cell membrane integrity, which was also verified by the principal component analysis (Figure 6).

The findings from this research experiment show that Cd-induced stress enhanced the levels of Cd in various parts of plant tissues (roots, shoots and seeds) in comparison with the control. However, the gradual addition of Si content restricted the translocation of Cd concentrations in plant tissues [1,2]. These findings indicate that Si treatment using various Si salts under Cd stress showed a significant reduction in Cd^2+^ to its optimum concentration in various plant tissues of peas. This demonstrates that Si restricted the uptake of Cd^2+^ by pea roots and limited movement into plant leaves [61]. Likewise, similar outcomes were also accounted for in alfalfa [56], maize (*Zea mays*) [61], wheat [2] and rice [1]. The rise in plant resistance to Cd^2+^ might be due to a decline in Cd^2+^ uptake and transport and to advances in plant resilience [62]. In this experiment, 1.00 mM of Si concentration notably reduced the Cd^2+^ accumulation levels in pea tissues in correlation with only Cd stress. The Cd^2+^ aggregation was much higher in plant roots as compared to seeds and shoots [63]. An Si-interceded decline in Cd^2+^ uptake or changes from roots to leaves has been suggested to add to the expansion of Cd^2+^ plant resistance [1,2,61]. Similar findings were also observed where Si addition in garlic (*Allium sativum*) plants decreased the Cd^2+^ accumulation in various parts of plants; nonetheless, the Cd^2+^ contents in roots were greater in comparison with other parts of plants, such as the shoot and the bulb [64]. Thus, Si can reduce Cd absorption in plant tissues not only by reducing metal bioaccumulation but by promoting plant productivity as well [65,66]. Therefore, the optimum level of Si using potassium silicate is the best option for limiting Cd uptake in plant tissue.

## 5. Conclusions

Cadmium stress reduced the growth, phenological and biomass-related traits and enhanced the Cd content in various tissues. The addition of Si to soils in an appropriate amount promoted growth and biochemical attributes by decreasing the electrolyte leakage and the Cd accumulation in pea plants. The efficacy of silicate compounds in mitigating Cd toxicity varies with its form, the type of chemical and the quantity of foliar application. The systematic change in decreasing trends in the plant growth, photosynthetic attributes and metal accumulation for the silicate compounds followed the order of K-silicate > Na-silicate, and for the foliar application treatment concentrations, the trend was 1 mM > 1.5 mM > 0.5 mM > 0 mM. The results depict that the foliar application (1.00 mM) was effective in enhancing the growth and health of peas while lowering Cd uptake and movement in pea plant tissues, which is a potential approach to manage and control the entry of Cd into the human body system through crops. Nevertheless, further field experiments should be seriously investigated to conclude a more precise and effective strategy in mitigating Cd stress under a diverse climate.

## Figures and Tables

**Table 1:** Effect of various silicate salts with various doses of foliar applications on the health risk assessment (HRI) values of adults and children of peas grown under normal and cadmium (Cd)-stressed conditions.

Experimental Treatments			HRI Adults	HRI Children
Control (0 mg kg^−1^)	Sodium Silicate	0.00 mM	0.11	0.20
		0.50 mM	0.08	0.15
		1.00 mM	0.03	0.05
		1.50 mM	0.05	0.10
	Potassium Silicate	0.00 mM	0.11	0.21
		0.50 mM	0.05	0.10
		1.00 mM	0.02	0.03
		1.50 mM	0.04	0.07
Cd Stress (20 mg kg^−1^)	Sodium Silicate	0.00 mM	0.51	0.95
		0.50 mM	0.42	0.79
		1.00 mM	0.35	0.65
		1.50 mM	0.45	0.83
	Potassium Silicate	0.00 mM	0.53	0.98
		0.50 mM	0.39	0.73
		1.00 mM	0.30	0.57
		1.50 mM	0.41	0.75

**Table 2:** Correlation matrix of growth, water-related, enzymatic and biochemical attributes of peas in response to various rates of foliar-applied silicon (0, 0.50 mM, 1.00 mM and 1.50 mM) under normal and Cd-stressed conditions.

Variables	CAT	CC	EL	LA	POD	PR	RDW	RWC	SC	SDW
CC	−0.72 **									
EL	0.85 **	−0.86 **								
LA	−0.46 **	0.65 **	−0.35 *							
POD	0.92 **	−0.77 **	0.87 **	−0.47 **						
PR	−0.78 **	0.90 **	−0.82 **	0.67 **	−0.83 **					
RDW	−0.73 **	0.94 **	−0.76 **	0.77 **	−0.74 **	0.89 **				
RWC	−0.68 **	0.87 **	−0.75 **	0.69 **	−0.72 **	0.87 **	0.89 **			
SC	−0.78 **	0.95 **	−0.91 **	0.61 **	−0.84 **	0.90 **	0.88 **	0.88 **		
SDW	−0.65 **	0.88 **	−0.71 **	0.66 **	−0.64 **	0.79 **	0.93 **	0.78 **	0.79 **	
SOD	0.89 **	−0.66 **	0.79 **	−0.41 **	0.91 **	−0.73 **	−0.65 **	−0.60 **	−0.76 **	−0.54 **

CAT = Catalase activity; CC = Chlorophyll content; EL = Electrolyte leakage; LA = Leaf area; POD = Peroxidase activity; RDW = Root dry weight; RWC = Relative water content; SOD = Superoxide dismutase activity; SC = Stomatal conductance; SDW = Shoot dry weight; ** = Significant at *p* ≤ 0.01; * = Significant at *p* ≤ 0.05.

## Data Availability

Not applicable.
